# Complex wavefront sensing based on coherent diffraction imaging using vortex modulation

**DOI:** 10.1038/s41598-021-88523-x

**Published:** 2021-04-27

**Authors:** Rujia Li, Liangcai Cao

**Affiliations:** State Key Laboratory of Precision Measurement Technology and Instruments, Department of Precision Instruments, Tsinghua University, Beijing, 100084 China

**Keywords:** Optics and photonics, Optical techniques, Imaging and sensing

## Abstract

Phase retrieval seeks to reconstruct the phase from the measured intensity, which is an ill-posed problem. A phase retrieval problem can be solved with physical constraints by modulating the investigated complex wavefront. Orbital angular momentum has been recently employed as a type of reliable modulation. The topological charge *l* is robust during propagation when there is atmospheric turbulence. In this work, topological modulation is used to solve the phase retrieval problem. Topological modulation offers an effective dynamic range of intensity constraints for reconstruction. The maximum intensity value of the spectrum is reduced by a factor of 173 under topological modulation when *l* is 50. The phase is iteratively reconstructed without a priori knowledge. The stagnation problem during the iteration can be avoided using multiple topological modulations.

## Introduction

Measuring the complex wavefront from recorded intensity images is demanded in various fields, including microscopy^1,2^, optical element characterization^3,4^ and imaging through scattering media^5,6^. Due to the extremely high frequency of light, only the intensity is directly recorded by available sensors. To acquire the phase, the investigated complex wavefront is modulated in amplitude, phase and polarization. For instance, modulated intensity fringes have been projected to measure the profile of the object^7,8^. Phase modulations have been used to reconstruct the measured complex wavefronts in a compact and robust setup^9,10^. The polarization of the illumination or object wavefront has been combined for high-resolution quantitative phase imaging^11,12^. Thus, the phase can be obtained from redundant measurements with modulations.

The coherent diffraction imaging (CDI) technique is a powerful and robust method to solve the phase retrieval problem. The complex wavefront is iteratively reconstructed from the measured intensities with a priori knowledge. In 1971, the Gerchberg-Saxton (G-S) algorithm was proposed, requiring strong constraints to work with measured intensities^13^. Then, Fienup modified the G-S algorithm by replacing the strong constraints with loose constraints^14,15^. The phase could be retrieved from the measured intensities with all kinds of constraints. Modulations can be constraints in CDI to retrieve the phase. In 2004, Rodenburg used shifting illumination in the CDI technique^16^. Then, the complex specimen can be reconstructed over a large field of view and at high resolution^17^. In 2007, F. Zhang used phase modulations to realize reconstruction without a priori knowledge^18^. Quantitative phase imaging was also achieved using intensity modulation^19^. In the aforementioned CDI works, the phase can be successfully recovered from the measured intensity with modulations.

The constraint is of vital importance to CDI systems. Traditional phase retrieval iterations suffer from the stagnation problem using a single intensity constraint. By introducing multiple random phase constraints^18^, uncorrelated terms are generated from different modulations. The stagnation in the iteration can be avoided to reach an optimal reconstruction. The uncorrelated terms need to be captured using an imaging sensor with a high signal-to-noise ratio (SNR). In our previous work, the structured phase constraint was designed to increase the SNR^9^. The uncorrelated terms were introduced by shifting the modulation pattern and changing the pattern’s direction. A robust and successful reconstruction was achieved.

Vortex beams carry orbital angular momentum (OAM) modes^20^. These modes are characterized by the topological charge *l*, a quantum number. Theoretically, this charge is unbounded in the free space. State space with an infinite dimension can be constituted by the combination of OAM modes. As an emerging modulation dimension, the topological charge robustly propagates when there is atmospheric turbulence^21,22^. Many breakthroughs have been reported based on novel topological modulations. High-speed and large-capacity optical communications have been achieved^23,24^. OAM-dependent holographic images were multiplexed to achieve high-security optical encryption^25^. The complex diffraction patterns of an unusual periodic object could be manipulated^26^. A bright solid-state laser source with high purity and collection efficiency was also invented^27^.

In this work, we utilize topological modulations to solve the phase retrieval problem. The complex wavefront is modulated with the vortex phase, which forms topological constraints. The spectrum intensities of the modulated wavefront are recorded for intensity constraints. The uncorrelated terms in the intensity constraints are provided by multiple topological modulations. The complex-amplitude object is iteratively reconstructed using topological and intensity constraints without a priori knowledge. The stagnation problem in the reconstruction iterations can be avoided with sufficient topological constraints. Unlike the simple phase modulation in our previous work^9^, the zeroth order and low frequency terms in the modulated spectrum are suppressed by the increasing topological charge. Therefore, the recorded intensity is spread and diffused in the Fourier spectrum domain, which offers an effective dynamic range of intensity constraints for the reconstruction.

## Modulating the complex-amplitude object

**Figure 1:**
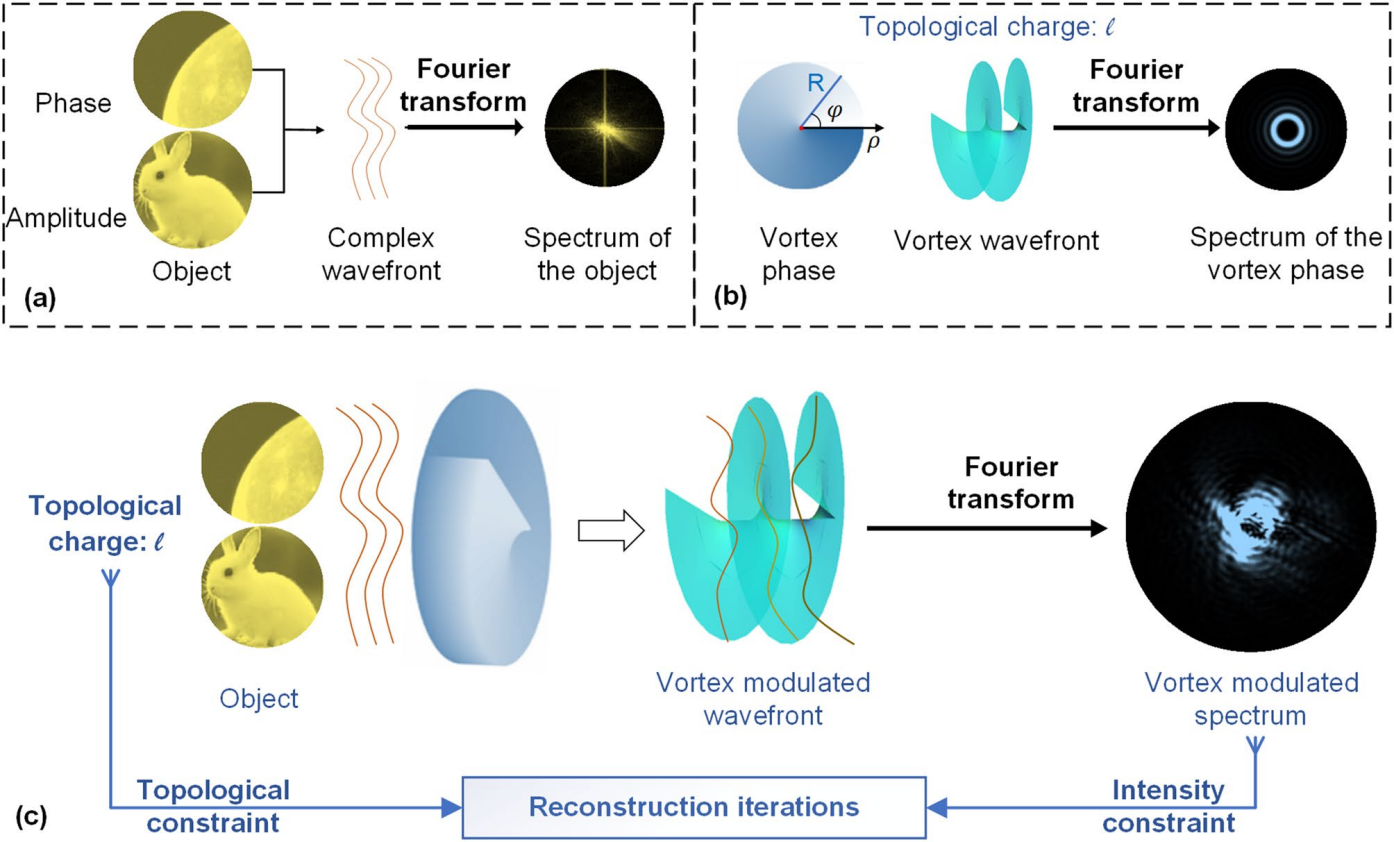
Schematic of the vortex modulation method. **(a)** Phase and amplitude of the complex-amplitude object and its Fourier spectrum, which are shown in grayscale images with yellow hue; **(b)** vortex phase with topological charge *l*; **(c)** the complex-amplitude object modulated with the vortex phase and its Fourier spectrum with topological modulation.

A general schematic diagram of the CDI with topological modulation is illustrated in Fig. 1. The investigated complex-amplitude object has amplitude and phase components. As shown in Fig. 1a, the amplitude component is an image of a rabbit, and the phase component is an image of the moon. Vortex phases with designed topological charge *l* are used for modulation. By changing the topological charge gradually, the spectrum intensities of the modulated wavefront are recorded sequentially. Spectrum intensities are the intensity constraints, and the designed topological charges are the topological constraints. The complex-amplitude object is iteratively reconstructed with both intensity constraints and topological constraints.

The investigated complex-amplitude object is expressed as $\widetilde{u}\left( {x,y} \right) = u\left( {x,y} \right)\exp \left[ {i\varphi_{u} \left( {x,y} \right)} \right]$. Thus, the Fourier spectrum is $\widetilde{U}\left( {f_{x} ,f_{y} } \right) = U\left( {f_{x} ,f_{y} } \right)\exp \left[ {i\varphi_{U} \left( {f_{x} ,f_{y} } \right)} \right]$; *x* and *y* and $f_{x}$ and $f_{y}$ are the Cartesian coordinates in the object domain and Fourier spectrum domain, respectively. The intensity of the spectrum $\left| {U\left( {f_{x} ,f_{y} } \right)} \right|^{2}$ is measured as the intensity constraint. It is impossible to reconstruct the complex-amplitude object $\widetilde{u}\left( {x,y} \right)$ directly from the intensity constraint due to the loss of the phase.

To solve the phase retrieval problem, topological constraints are introduced. The topological modulations are applied by the vortex phase, of which the transmission function $\widetilde{v}_{l} \left( {r,\varphi } \right)$ can be described in polar coordinates as:

1 $$ \widetilde{v}_{l} \left( {r,\varphi } \right) = circ\left( \frac{r}{R} \right)\exp \left( {il\varphi } \right), $$

where *r* and $\varphi$ are respectively the radius and azimuthal angle in the polar coordinates, *l* is the topological charge of the vortex phase, *R* is the geometric radius of the vortex phase modulation, and *circ*() depicts the circular function. The phase profile of a vortex modulation is increased continually along a closed phase loop from 0 to $2\pi l$^28^. The central point in the vortex phase is the phase singularity. If there is a tiny circle *S* around the phase singularity, the topological charge *l* is defined as the number of phase loops from 0 to 2 $\pi$. The topological charge is

2 $$ l = \frac{1}{2\pi }\oint\limits_{S} {\Delta \varphi dS}, $$

where $\Delta \varphi$ is the phase variation along the arc length $dS$. The spectrum $\widetilde{V}_{l}$ of the vortex phase $\widetilde{v}_{l}$ can be derived from the Fourier transform in polar coordinates,

3 $$ \widetilde{V}_{l} \left( {\rho ,\theta } \right) = \mathcal{F}\left\{ {\left. {\widetilde{v}_{l} \left( {r,\varphi } \right)} \right\}} \right. = 2\pi \left( { - 1} \right)^{l + 1} \exp \left( {il\theta } \right)\int_{0}^{R} {J_{l} \left( {2\pi r\rho } \right)rdr} ,$$

where $\mathcal{F}\left. {\left\{ {} \right.} \right\}$ depicts the Fourier transform and $\rho$ and $\theta$ are the polar coordinates in the spectrum domain. $J_{l} \left( {} \right)$ represents the *l*th-order Bessel function. Considering the intensity of the spectrum, the modulus amplitude of $\widetilde{V}_{l}$ is

4 $$ \left| {\widetilde{V}_{l} \left( \rho \right)} \right|^{2} = \left| {2\pi \int_{0}^{R} {J_{l} \left( {2\pi r\rho } \right)} rdr} \right|^{2} .$$

The intensity distribution of $\widetilde{V}_{l}$ is related to the integral of the *l*th-order Bessel function, which is radially symmetric. When the vortex phase is applied, the topological charge *l* is not zero. In the center of the spectrum ($\rho =0$) of the vortex phase, there is a black hole ($\left| {\widetilde{V}_{l} \left( 0 \right)} \right|_{l \ne 0}^{2} = 0$). The intensity of $\widetilde{V}_{l}$ has a ring shape, as shown in Fig. 1c. While the topological charge increases, the helical vortex phase becomes more twisted, and the radius of the ring in the spectrum domain increases.

The measured complex-amplitude object is modulated with the vortex phase. Topological modulation is analyzed based on angular spectrum theory. For the complex-amplitude object $\tilde{u}$ modulated by the vortex phase $\tilde{v}_{l}$, the modulated wavefront is $\tilde{u}_{l}$, $\tilde{u}_{l} = \tilde{u} \times \tilde{v}_{l}$. The spectrum of $\tilde{u}_{l}$ can be derived from the convolution of the spectrum of the complex-amplitude object and vortex modulation. The intensity of the spectrum is,

5 $$ \left| {\widetilde{U}_{l} } \right|^{2} = \left| {\left[ {\widetilde{U}\left( {f_{x} ,f_{y} } \right)} \right] \otimes \left[ {\widetilde{{V_{l} }}\left( {\rho ,\theta } \right)} \right]} \right|^{2}, $$

where the coordinates can be converted as $\rho = \sqrt {f_{x}^{2} + f_{y}^{2} } ,\;\theta = \arctan \frac{{f_{y} }}{{f_{x} }}$.

## Spectrum with vortex modulation

**Figure 2:**
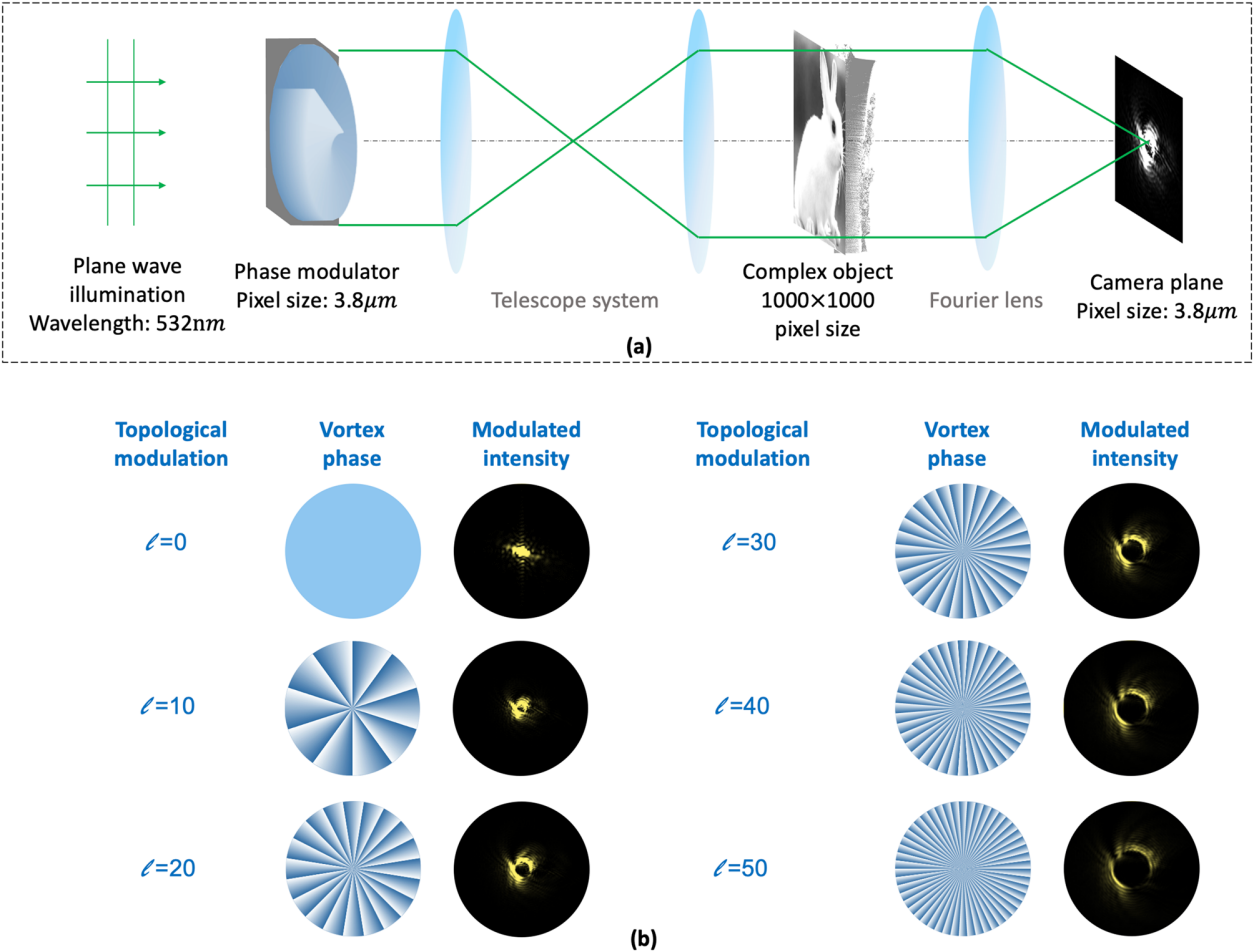
**(a)** Schematic of the coherent diffraction imaging system with topological modulations. **(b)** Vortex phases on the phase modulator and modulated intensities with topological charge *l* from 0 to 50 at an interval of 10.

A simulation is presented to study the CDI system with vortex modulations. The schematic is shown in Fig. 2a. The input plane wave has a wavelength of 532 nm. The size of the complex-amplitude object is 1000 × 1000 pixels. The amplitude value of the complex-amplitude object is normalized from 0 to 1, and the phase value is from 0 to $\pi$. The modulator plane is conjugated to the object plane using a telescope system. The investigated complex-amplitude object can be modulated with the vortex phase. Then, the modulated wavefront is Fourier transformed. The spectrum intensity is recorded by a sensor with a pixel size of 3.8 $\mathrm{\mu m}$. Six vortex phases are utilized for modulation, of which the topological charge *l* ranges from 0 to 50 at an interval of 10. The vortex phase and the corresponding spectrum intensities are shown in Fig. 2b.

The spectrum is modulated with topological charge *l* in two aspects. The first is the intensity distribution of the modulated spectrum. As shown in Fig. 2, when *l* = 0 and there is no modulation, the intensity represents the spectrum intensity of the complex-amplitude object. The frequency terms are concentrated in the central area of the spectrum. As *l* is gradually increased from 10 to 50, the vortex modulation brings a dark hole into the modulated spectrum. This is because the modulated spectra come from the spectrum convolution as derived in Eq. (5). The spectrum of the vortex phase is ring-shaped, and the radius increases with increasing *l*. The modulated spectrum is distributed along the ring-shaped spectrum of the vortex phase. With increasing topological charge *l*, scanning along the radius direction of the ring is performed in the spectrum domain.

**Figure 3:**
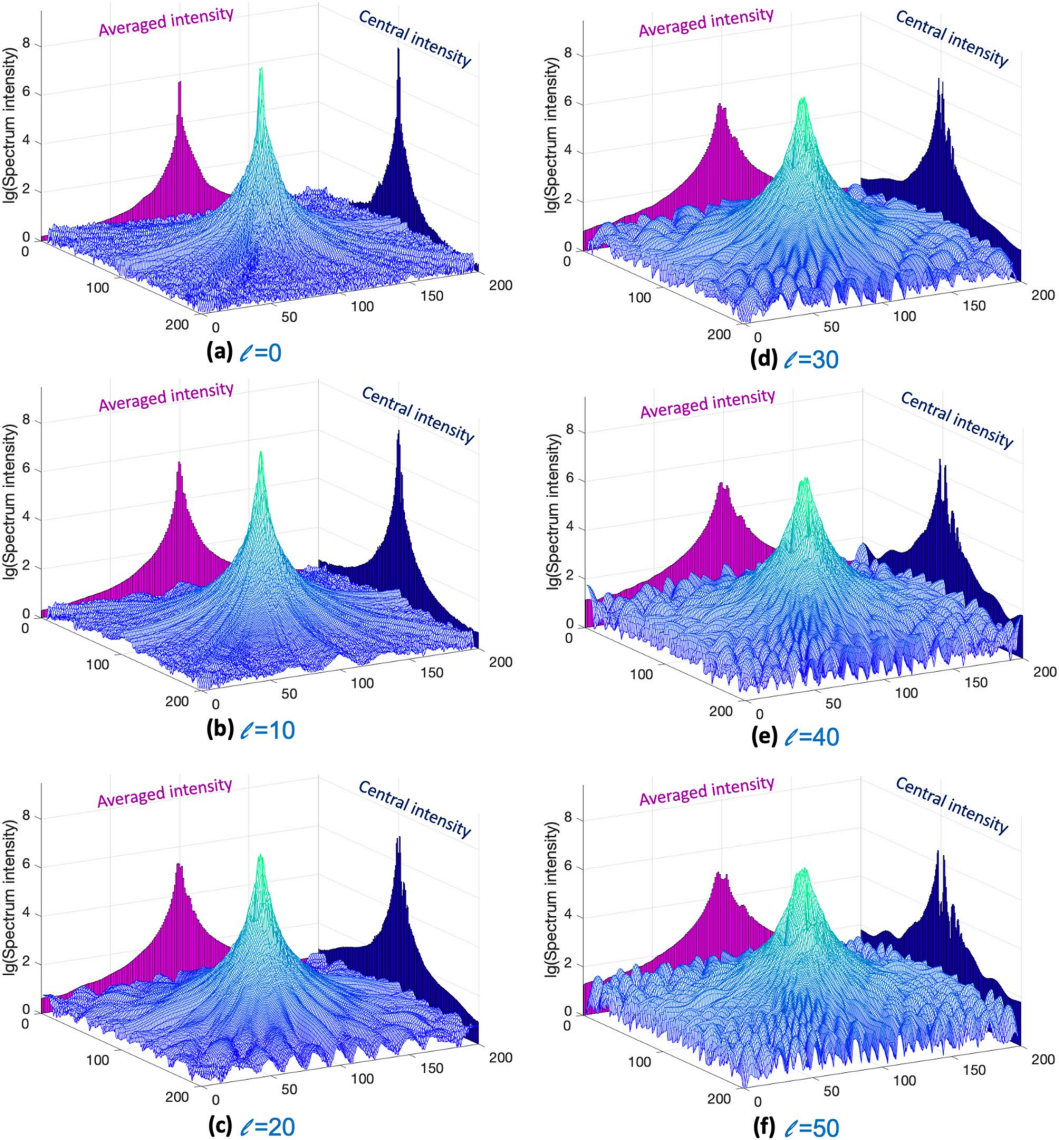
3D profile of the logarithmic intensity values of the modulated spectrum with topological charge *l* from 0 to 50 at an interval of 10. The averaged intensity is the averaged value along the line. The central intensity is the intensity value along the central line. **(a)** *l* = 0; **(b)** *l* = 10; **(c)** *l* = 20; **(d)** *l* = 30; **(e)** *l* = 40; **(f)** *l* = 50.

Second is the spread of the energy in the spectra*.* For better visualization, the 3D profile of the modulated spectrum is shown in Fig. 3. The sizes of the profile are down-sampled to 200 × 200 pixels using the bicubic interpolation method. When *l* is zero, there is no topological modulation. The energy of the spectrum is concentrated in the zeroth-order term and low-frequency terms. The intensity values of the high-frequency terms are far smaller than those of the low-frequency terms. The high intensity is greater than the low intensity by five orders of magnitude. When topological modulations are applied and *l* is increasing, the energy of the spectrum is spread in the radial direction from zeroth order. Based on the principle of energy conservation, the intensity values of the low-frequency terms are decreased, and those of the high-frequency terms are increased. Now, the high intensity is greater than the low intensity by three orders of magnitude.

**Figure 4:**
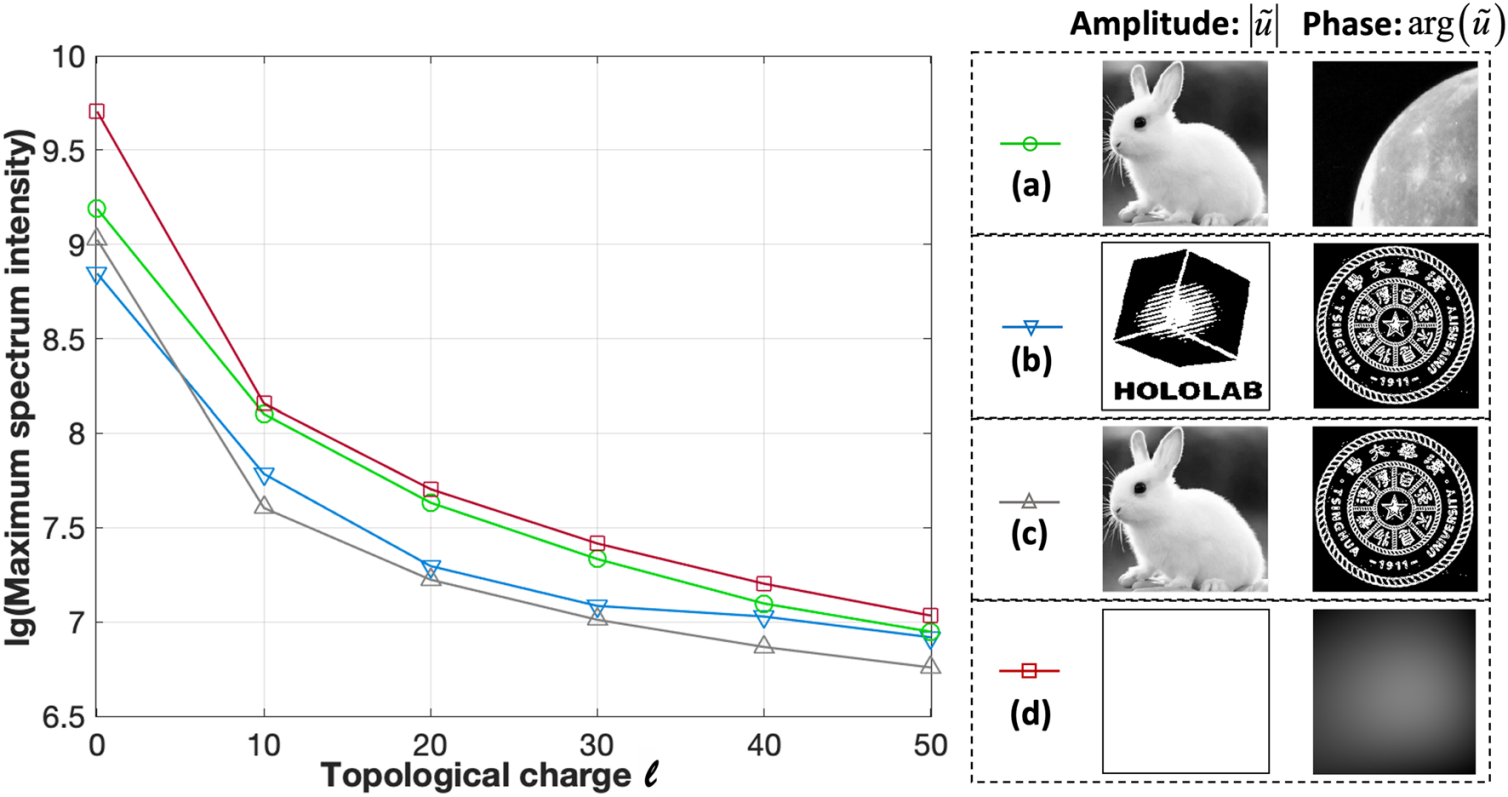
Logarithmic profile of the maximum intensity value of the modulated spectrum with topological charge *l* from 0 to 50. **(a)** Amplitude and phase; **(b)** binary amplitude and binary phase; **(c)** amplitude and binary phase; **(d)** wavefront. arg(): phase operator.

The maximum intensity value of the modulated spectrum for different topological charges *l* is shown in Fig. 4. Four complex-amplitude objects are investigated. Figure 4a–c show the amplitude and phase of the object in different grayscale or binary modes. Figure 4d shows the wavefront. The maximum intensity values of all the spectra are modulated to decrease more than 100 times with increasing topological charge. Taking the complex-amplitude object (a) as an example, when *l* = 0, the maximum intensity value of the original spectrum is 10^9.18^. As *l* is increased from 0 to 50, the maximum intensity decreases rapidly. When *l* is 50, the maximum intensity value is 10^6.94^, representing a decrease by a factor of 173.

## Reconstruction with topological constraints

**Figure 5:**
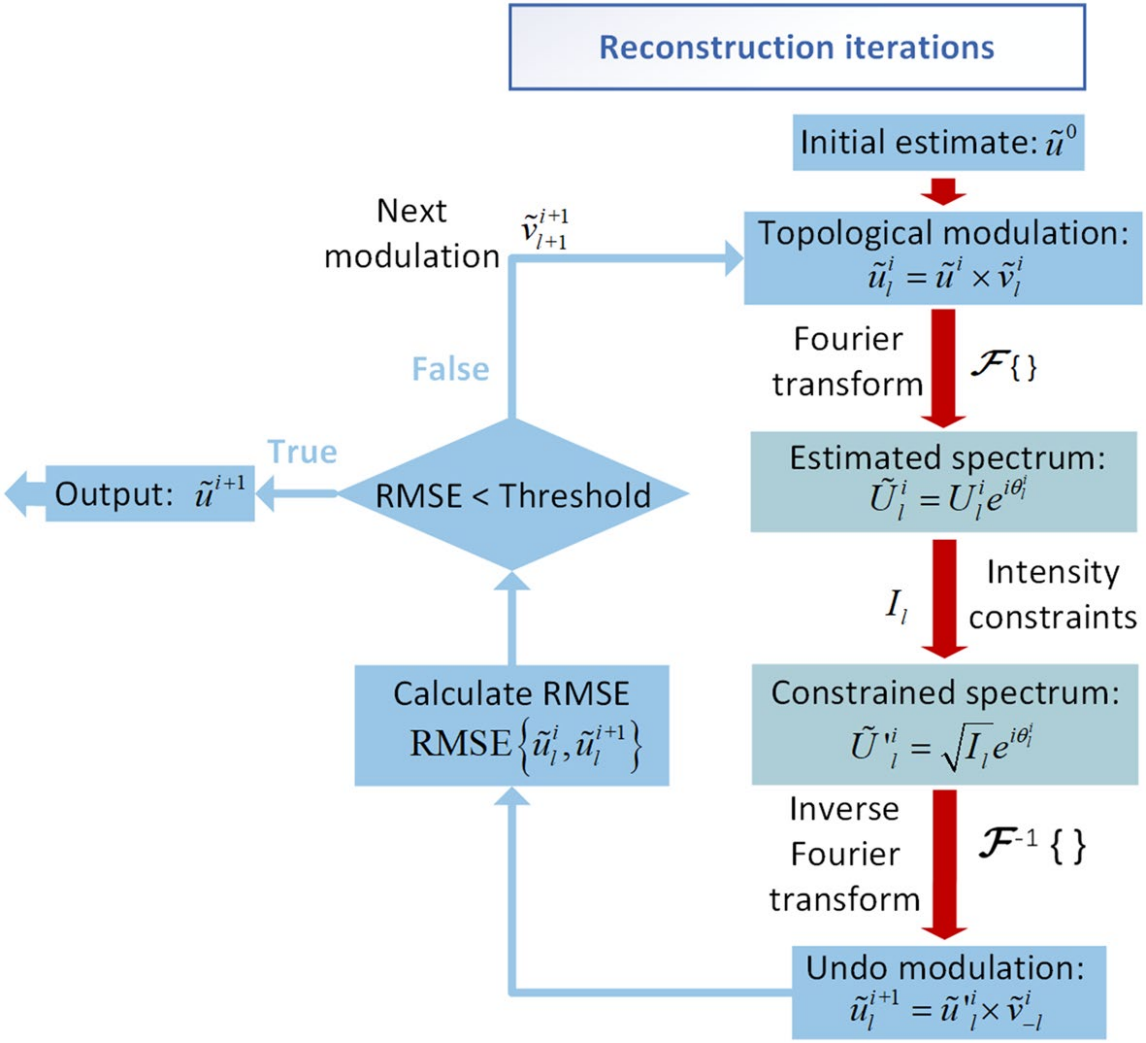
Flowchart of the reconstruction iteration algorithm using topological modulations.

Reconstructing the complex-amplitude object from the measured intensity is an ill-posed problem. Multiple modulations and redundant data can be utilized to solve this problem. In this work, multiple vortex phases are modulated to form topological constraints. The spectrum intensities are recorded to form intensity constraints. The complex-amplitude object is iteratively reconstructed with both topological constraints and intensity constraints. The iteration algorithm is shown in Fig. 5. The iteration starts with a random initial guess $\tilde{u}^{0}$ of the complex-amplitude object, and $\tilde{u}^{0}$ is modulated with the vortex phase $\tilde{v}_{l}$. Then, the modulated complex wavefront $\tilde{u}^{0} \times \tilde{v}_{{_{l} }}$ is Fourier transformed to obtain the guess of the modulated spectrum $\tilde{U}^{0}_{l}$. The intensity constraint is applied by replacing the amplitude of $\tilde{U}^{0}_{l}$ with the measured intensity. The constrained spectrum is inversely Fourier transformed to refresh the modulated wavefront. The topological constraint is applied by removing the vortex phase from the refreshed modulated wavefront. At the end of this iteration loop, the guess of the complex-amplitude object $\tilde{u}^{1}$ is acquired with one pair of topological and intensity constraints. After one iteration loop, other pairs of constraints are sequentially applied.

As the iteration proceeds, the complex wavefront is reconstructed gradually. The iteration can be terminated after a predetermined number of iterations or reaching an acceptable error between two iteration loops. The error is evaluated by the root mean square error (RMSE),

6 $$ {\text{RMSE = }}\sqrt {\frac{1}{M \times N}\sum\limits_{x} {\sum\limits_{y} {\left| {u^{i + 1} \left( {x,y} \right) - u^{i} \left( {x,y} \right)} \right|^{2} } } } $$

where the size of the matrix is *M* × *N* pixels. The amplitude and phase components of the complex wave front should be evaluated by the RMSE.

Phase retrieval is a nonlinear ill-posed problem. Two aspects are essential to successful phase retrieval: (a) the pairs of constraints should outnumber the unknowns; (b) uncorrelated terms are required to make effective constraints. Theoretically, there are three unknowns, including the amplitude and phase in the object plane and the phase in the recording plane. Four or more pairs of constraints are needed for successful reconstruction. In this simulation, six pairs of constraints are utilized to obtain better iteration convergence. The intensity constraints with varying modulations should be different to introduce the uncorrelated terms. Multiple vortex phases are generated with different topological charges. Scanning in the radical direction is performed in the spectrum intensities. Pixels engaged to record the spectrum intensities are significantly different before and after the modulation.

**Figure 6:**
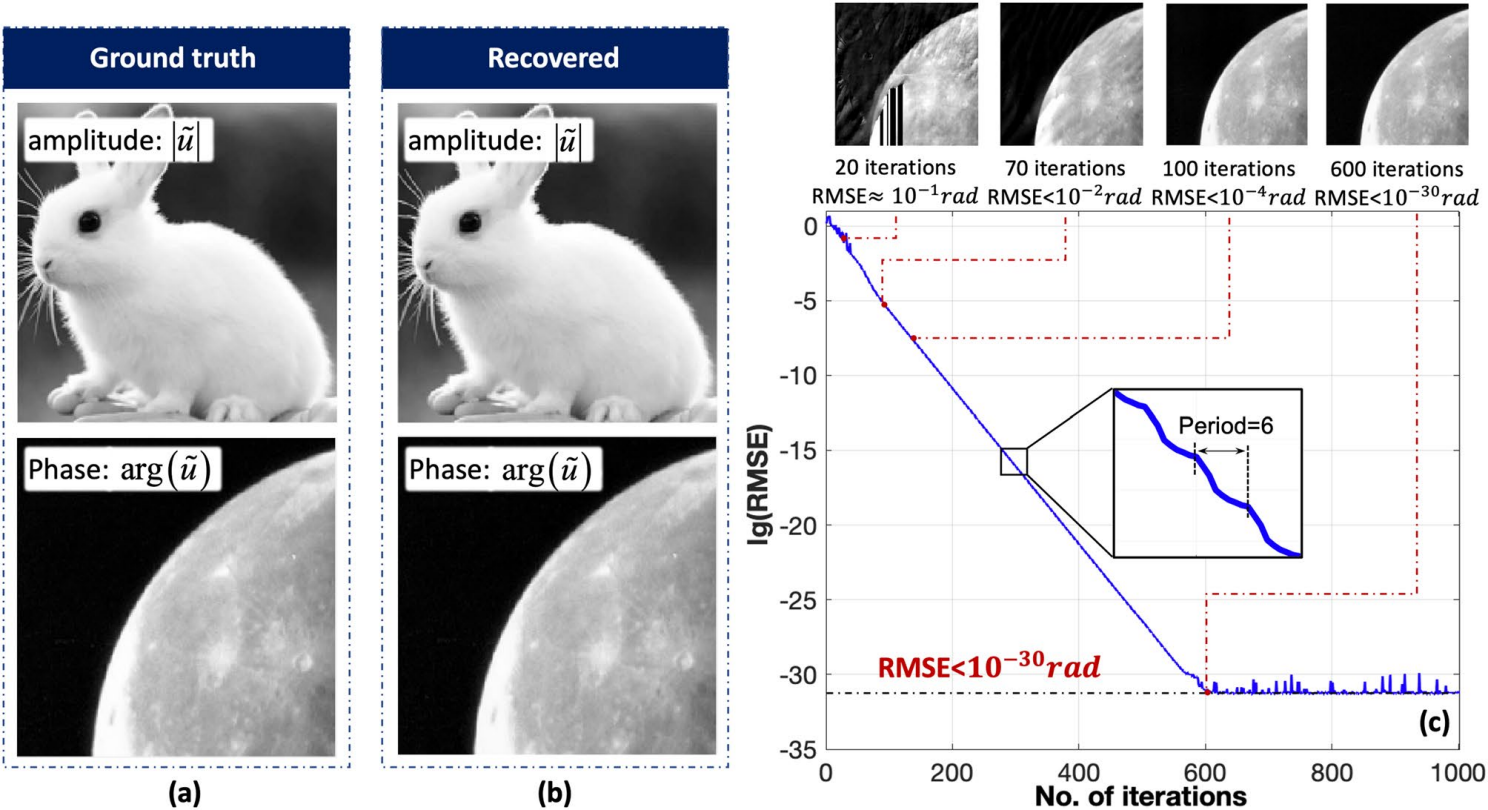
**(a)** Ground truth of the investigated complex-amplitude object. **(b)** Recovered complex-amplitude object using six topological modulations after 1000 iterations. **(c)** lg(RMSE) between the ground truth and recovered phase for iteration numbers from 0 to 1000. arg(): phase operator.

Figure 6 presents the reconstructions when the iteration number is from 0 to 1000. With topological constraints and intensity constraints, the calculation starts to converge as the iteration number is increased. After 20 iterations, the outline of the phase can be distinguished. After 70 iterations, the artifacts on the phase are eliminated. As the iteration number is increased from 70 to 600, the RMSE decreases. The RMSE fluctuates slightly at the period of six iterations because the number of constraints was also six. Finally, the calculation enters the final stage when the iteration number is above 600. The reconstruction of the complex-amplitude object reaches the optimal estimate under current constraints. The RMSE between the fully optimized phase and the ground truth is approximately $2.8\times {10}^{-31}$ rad. The RMSE of the reconstructed amplitude is near $1.08\times {10}^{-31}$ rad, which shows the high performance of the topological modulation method in coherent diffraction imaging.

## Conclusion

This work explores a new modulation dimension in coherent diffraction imaging. Topological modulations are utilized to solve the phase retrieval problem. High-quality reconstruction can be achieved with six pairs of topological constraints. Based on the features of the vortex phase, the dynamic range of spectrum intensities is physically reduced. The maximum value of the intensity is reduced by a factor of 173 when *l* is 50. The example shown here paves the way for using vortex phase modulation for imaging in the spectrum domain with a high signal-to-noise ratio. In addition to traditional modulations, OAM can be an additional degree of freedom in the field of computational imaging.
